# MiRenSVM: towards better prediction of microRNA precursors using an ensemble SVM classifier with multi-loop features

**DOI:** 10.1186/1471-2105-11-S11-S11

**Published:** 2010-12-14

**Authors:** Jiandong Ding, Shuigeng Zhou, Jihong Guan

**Affiliations:** 1 Shanghai Key Lab of Intelligent Information Processing, and School of Computer Science, Fudan University, Shanghai 200433, China; 2 Department of Computer Science and Technology, Tongji University, Shanghai 201804, China

## Abstract

**Background:**

MicroRNAs (simply miRNAs) are derived from larger hairpin RNA precursors and play essential regular roles in both animals and plants. A number of computational methods for miRNA genes finding have been proposed in the past decade, yet the problem is far from being tackled, especially when considering the imbalance issue of known miRNAs and unidentified miRNAs, and the pre-miRNAs with multi-loops or higher minimum free energy (MFE). This paper presents a new computational approach, miRenSVM, for finding miRNA genes. Aiming at better prediction performance, an ensemble support vector machine (SVM) classifier is established to deal with the imbalance issue, and multi-loop features are included for identifying those pre-miRNAs with multi-loops.

**Results:**

We collected a representative dataset, which contains 697 real miRNA precursors identified by experimental procedure and other computational methods, and 5428 pseudo ones from several datasets. Experiments showed that our miRenSVM achieved a 96.5% specificity and a 93.05% sensitivity on the dataset. Compared with the state-of-the-art approaches, miRenSVM obtained better prediction results. We also applied our method to predict 14 Homo sapiens pre-miRNAs and 13 Anopheles gambiae pre-miRNAs that first appeared in miRBase13.0, MiRenSVM got a 100% prediction rate. Furthermore, performance evaluation was conducted over 27 additional species in miRBase13.0, and 92.84% (4863/5238) animal pre-miRNAs were correctly identified by miRenSVM.

**Conclusion:**

MiRenSVM is an ensemble support vector machine (SVM) classification system for better detecting miRNA genes, especially those with multi-loop secondary structure.

## Background

MicroRNAs (miRNAs) [1] are single-stranded, endogenous ~22nt small non-coding RNAs (sncRNA) that can play important regular roles in animals and plants by targeting mRNA for cleavage or post-translation repression [2]. Mature miRNAs are derived from longer precursors (pre-miRNAs), each of which can fold into a hairpin structure that contains one or two mature miRNAs in either or both its arms. Accordingly, miRNA biogenesis is highly regulated, controlled at both transcriptional and post-transcriptional levels [3], and overexpression and underexpression of miRNAs are linked to various human diseases, particularly cancers [4,5].

MiRNAs are always located in the introns of protein-coding genes [6], introns and exons of non-coding genes [7]. In mammalian genomes, it is also possible to find miRNAs in repetitive regions, and some studies suggest that transposable elements may be involved in the creation of new miRNAs [8]. MiRNA biogenesis in animals contains two steps [2]. In the first step, the primary miRNA (pri-miRNA), which is several hundred nucleotides long, is processed in the nucleus by a multiprotein complex containing an enzyme called *Drosha* to produce the ~70nt long miRNA stem-loop precursor (pre-miRNA), which is then exported to the cytoplasm. In the cytoplasm, the second step takes place where the pre-miRNA matures into a ~22nt long miRNA:miRNA* duplex, with each strand originating from opposite arms of the stem-loop [9]. Then, the miRNA strand of the miRNA:miRNA* duplex is loaded into a ribonucleoprotein complex known as the miRNA-induced silencing complex (miRISC). Until recently, the miRNA* was thought to be peeled away and degraded. However, some studies indicate that miRNA* is also sorted into Argonauts and might have a regular function in Drosophila melanogaster [10,11].

Identification of miRNA genes is an eminent and challenging problem towards the understanding of post-transcriptional gene regulation. The short length of miRNAs and their ability to act redundantly or to have only a subtle phonotypical impact impose a limitation to the use of mutagenesis and other conventional genetics techniques [12]. Direct cloning is the initial choice, but only abundant miRNA genes can be easily detected. Since not all miRNAs are well expressed in many tissues, miRNAs that have very low expression levels or that are expressed tissue-specifically possibly can not be detected, and recently research suggests that lowly expressed Human miRNA genes evolve rapidly [13]. This situation is partially mitigated by the deep-sequencing techniques that nevertheless require extensive computational analysis to distinguish miRNAs from other small non-coding RNAs of the same size [14]. Therefore, computational approaches are essential for miRNA gene finding in sequenced genomes.

In these years, large-scale computational approaches have been developed, such as filter-based approaches [6,15], homology-based research [16,17], mixed approaches [14,18], and machine learning methods. Filter-based approaches (e.g. *MirScan, mirSeeker*), focusing on identifying high-quality sets of conserved miRNA candidates, are able to recover a substantial part of the known miRNAs. However, they are critically dependent on conservation criteria to obtain reasonable specificity. Homology-based approaches (e.g. *ERPIN, MiRAlign*) rely exclusively either on sequence conservation or structure conservation so that lineage- or species-specific miRNA genes may escape the detection. In fact, many miRNA gene prediction approaches incorporate a homology search as part of their protocols, in addition to the ordinary search for orthologous. Mixed approaches (e.g. *PalGrade, miRDeep*) combine experimental with computational procedures in order to identify a wider range of miRNAs. As mentioned above, experimental approaches cannot easily detect low-expression or tissue-specific miRNAs.

The most popular computational miRNA gene finding methods are machine learning based approaches. Most of them share the same overall strategy but use different approaches to identify good stem-loop candidates, since they all try to generalize a positive set of already known miRNAs and a negative set of stem-loops that are not pre-miRNAs [19]. Several machine learning methods have been proposed to tackle the problem of identifying miRNA genes. SVM is a popular framework used to learn the distinctive characteristics of miRNAs. There are other machine learning methods that employ techniques such as HMM (Hidden Markov Model) [20,21], Random Forests [22], Naïve Bayes classifier [23], and Random walk algorithm [24] etc. Most approaches use sets of features including sequence conservation [25-27], topological properties [26,28], thermodynamic stability [26,27], and some other properties like entropy measures [27].

However, there are two major drawbacks with the existing machine learning based miRNAs identification approaches. One drawback is raised by the imbalance of positive and negative examples used. Since the real number of miRNAs in any given genome is still an open problem, it is assumed that there is a very few miRNA precursors in any randomly chosen stem-loop extracted from the genome. Positive examples are usually selected from miRNAs identified by experimental procedures or other computational methods. And the number of positive examples we can obtain is substantially smaller than that of negative examples. The imbalance issue between positive and negative examples can greatly degrade the performance of current machine learning approaches. Certainly, with a growing number of miRNAs being identified, we can expect an increasingly better performance from these methods. The other drawback lies in the fact that most existing machine learning based methods [23-25] make a few structural assumptions concerning stem length, loop size and number, as well as minimum free energy (MFE). Therefore, sequences with multi-branched loops secondary structure or MFE higher than -16 kal/mol possibly can not be predicted by those methods, which subsequently degrade the prediction performance. We have investigated Homo sapiens miRNAs in miRBase [29], and found that there are an increasing number of pre-miRNAs, which do not satisfy the above-mentioned assumptions (see Table S1 and S2 in the Additional file 1 for detail).

In this paper, we still treat the miRNA gene finding problem as a classification problem, and develop a powerful classification system, named miRenSVM, to overcome the two drawbacks mentioned above. On one hand, miRenSVM uses ensemble learning to deal with the imbalance issue; On the other hand, in addition to the features exploited by the existing methods, miRenSVM further includes the multi-loop features in its classifiers, and *F*-score is used to select final classification features. As a result, miRenSVM can achieve better performance than the existing methods.

In summary, miRenSVM distinct itself from the existing methods at least in three aspects: (1) Lower expression and tissue-specific miRNAs can be easily identified since different types of features are use. (2) Due to using ensemble SVM classifiers, both positive and negative examples can be exploited as many as possible. (3) No structural assumption for miRNA candidates is made. Particularly, multi-loop features are considered.

## Results

### Results of different features sets

We used 65 local and global features that are subsumed into three groups, which capture miRNA's sequence, secondary structure and thermodynamic properties respectively. In this section, we used single SVM classifier to check how different feature sets impact classification performance.

First, we trained a single SVM classifier with the entire training dataset to examine prediction performance by using each of the three features group separately. The classification performance is evaluated by the outer 3-fold cross validation method, which has been described in the method section. The results are listed in Table 1. Among the three feature subsets, the *base pair* group gets the highest SE (87.38%), while the *thermodynamic* group archives the best SP (98.99%), G_m_ (92.84%) and Acc (97.59%). The *triplet elements* group obtains a good SP (98.39%), but its SE is only 74.93%, which is much lower than that of the other two groups. From Table 1, we can see that: 1) Thermodynamics features are more discriminative than structure and sequence features in identifying miRNA precursors. Similar result was also obtained in [30]. 2) Base pair features are more useful in predicting real pre-miRNAs, since base pair group gets the highest *sensitivity*. 3) The four multi-loop features introduced in miRenSVM are effective in predicting pre-miRNAs with multi-loops, considering that nearly 84% pseudo pre-miRNAs and 4.76% real pre-miRNAs have secondary structure with multi-loops.

**Table 1:** Classification results obtained by outer 3-fold cross validation with different feature groups and feature selection

Feature Group	num	SE(%)	SP(%)	Gm(%)	Acc(%)
*triplet element*	32	74.93	98.39	85.87	95.64
*base pair*	15	**87.38**	98.24	92.65	97.00
*thermodynamics*	18	87.07	**98.99**	**92.84**	**97.59**

*all features*	65	87.50	98.82	92.99	97.47
*Selected by F-score*	32	87.78	98.88	93.16	97.58

Second, all the 65 features were used to train a single SVM classifier with the whole training dataset, and the performance was also evaluated by the outer 3-fold cross validation method. The results are SE (87.50%) and G_m_ (92.99%), which are a little better than the best results of using any individual features group. This indicates that the combination of different kinds of features can improve classification performance. The next step is to improve the prediction speed without degrading the accuracy rate. We thus considered feature selection method to select the intrinsic ones from all the 65 features. Feature selection is often applied to high dimensional data prior to classification learning. This procedure can reduce not only the cost of recognition by reducing the number of features that need to be collected, but in some cases it can also provide a better classification accuracy due to the finite sample size effect [31]. Here, we used *F*-score to select the best feature subset for our miRenSVM. This procedure is implemented by the *libsvm*'s feature selection tool. We evaluated the effectiveness of the feature subset selected by *F*-score method by training a single SVM classifier on the entire training set, and studying the sensitivity and the number of correctly predicted miRNAs. All the results of these experiments are summarized in Table 1. As shown in Table 1, after feature selection, the classification performance becomes better.

At last, the 32 features with the largest *F*-scores were used to train the miRenSVM classifier. This feature set contains 8 features from the *triplet elements* group, 8 features from the *base pair* group and 16 features from the *thermodynamic* group. Experimental results show that the 32 selected features subset not only obtains the highest classification results, but also greatly reduces the outer and inner cross-validation training time taken by SVM ensembles, especially when conducting class imbalance learning experiments presented in the next section. Table 2 lists all features used in the final SVM ensembles.

**Table 2:** 32 features selected by *F*-score

Group	num	Feature
*triplet element*	8	*A(((, A…, U(((, U(.(, U…, G(((, C(((, C(.(*
*base pair*	8	*dP, dP/n_loops, Avg_bp_stem, diversity, |A-U|/L,|G-C|/L, %(A-U)/n_loops, %(G-C)/n_loops *
*thermodynamics*	16	*NEFE, MFEI_1_, MFEI_2_, MFEI_3_, MFEI_4_, dG, Diff, Freq, Tm, dH/L, dS/L, Tm/L, p-value_MFE, p-value_EFE, z-score_MFE, z-score_EFE*

### Results of SVM ensembles

In this section we will present the experimental results of our miRenSVM approach. Two schemes, *majority vote* and *mean distance* (detail was delayed to the method section) were applied to aggregating the results of each sub SVM classifier. Since the ratio of negative samples to positive samples is 7.79:1, the cases of *k*=1, 2, 3, 4 or 8 were tested, respectively. We found that sub SVM classifiers trained with negative samples which are closer to the positive samples always achieve a lower SE than the other cases. And we called these datasets “closer set”. With this observation, we perform *majority vote* when even number of sub classifiers are employed in our miRenSVM. That is, when *k* is even (e.g. 2, 4, or 8) and the test sample receives equal numbers of positive and negative votes, the latter half of sub SVM classifiers takes priority over the former half trained with closer sets. Here, all experiments were conducted through the outer 3-fold cross validation method. Table 3 presents the average classification results of some SVM ensembles experiments.

**Table 3:** Results of classifier ensembles with different aggregation methods

Method	SE(%)	SP(%)	Gm(%)	Acc(%)
*majority vote(k=3)*	97.23	92.10	94.63	92.70
*majority vote(k=8)*	**97.91**	91.08	94.44	91.89
*mean distance(k=3)*	93.05	96.50	**94.76**	96.10
*mean distance(k=4)*	90.55	**97.79**	94.10	**97.91**

For each aggregation method, only the best two results are presented.

As shown in Table 3, both *majority vote* and *mean distance* get a better performance than using a single SVM classifier developed with the 32 selected features (G_m_ =93.16%). Compared with *mean distance* method, *majority vote* always archives higher *sensitivity* (SE), but its *specificity* (SP) is much lower, which impacts its overall *accuracy* (Acc). If this type of classifier is used for real-life prediction, due to its lower specificity, the chance of incorrectly predicting random sequences with stem-loop like secondary structure would be quite high. Therefore, we choose the best classifier developed under the *mean distance* method as the final miRenSVM classifier. The *mean distance* method obtains the best classification results on our dataset, that is, the highest G_m_ (94.76%) with SE=93.05% and SP=96.5%, and an acceptable Acc (96.1%). There is another reason to choose *mean distance*, that is efficiency. The ensemble SVM classifier predicts each test sample only one time while each test sample has to be predicted *k* times under *majority vote*.

We then validated our miRenSVM on the testing dataset. This set contains 14 Homo sapiens and 13 Anopheles gambiae miRNA precursor sequences newly published in miRBase13.0. The result shows that miRenSVM obtains 100% accuracy. Particularly, 4 sequences (MI0009983, MI0009988, MI0010486, and MI0010488) in the testing set whose MFE is higher than -13.70 kal/mol are all predicted correctly by our miRenSVM. In order to further demonstarte the advantage of the miRenSVM approach, we tested our miRenSVM on the miRBase13.0 and achieved a high sensitivity. MiRBase13.0 contains 27 animal genomes, including 5238 miRNA precursor sequences (not including *hsa* and *aga* pre-miRNAs). MiRenSVM correctly classified 92.84% (4863/5238) pre-miRNAs.

### Results of comparison with existing methods

**Figure 1:**
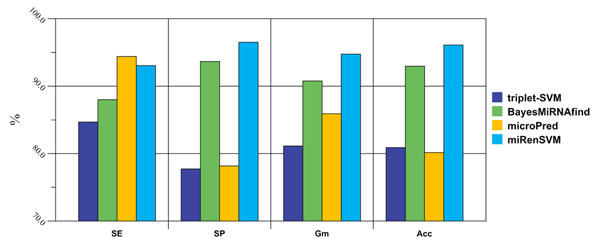
**Comparison between miRenSVM with other methods**. Three representative computational miRNA prediction methods are used to compare with our miRenSVM. *MicroPred* achieves the highest SE (94.4%), while miRenSVM gets the highest SP (96.5%), G_m_ (94.8%), and Acc(96.1%). The results are obtained by predicting 2060 sequences (250 real and 1810 pseudo pre-miRNAs).

We compared our approach with three existing methods that also used machine learning techniques to predict miRNA precursors [23, 25, 32] . These three compared methods include *triplet-SVM*, *BayesMiRNAfind* and *microPred*. The results of these methods are obtained by predicting 2060 sequences (250 real and 1810 pseudo pre-miRNAs) that have been already used in developing our MiRenSVM. This dataset contains two parts: 1/3 training set (223 real and 1810 pseudo pre-miRNAs) and the smaller testing set (27 bran-new *hsa* and *aga* pre-miRNAs). The results of these experiments are illustrated in Figure 1.

*triplet-SVM* was proposed by Xue *et al.*[25] to recognize pre-miRNAs based on the *triplet element* structure-sequence features. The method is trained on known human pre-miRNAs and obtains a high accuracy (~90%) when applied to several other species. Unlike miRenSVM, *triplet-SVM* uses only structure-sequence information, and therefore can predict miRNAs quickly. However, this method is not designed to detect miRNAs with multi-loop secondary structure or miRNAs with high MFE. *triplet-SVM* predicts only 518 (235 real and 283 pseudo) sequences. Although it has an acceptable sensitivity (84.68%), its specificity (77.74%) is not comparable to ours (96.5%).

*BayesMiRNAfind* was developed by Yousef *et al.*[23], which uses a different machine learning method, naïve Bayes classifier, to predict miRNAs conserved between human and mouse. Yousef *et al.* applied their method to the forward strand of the mouse genome sequence and present results for different score cut offs. *BayesMiRNAfind* is trained with cross-species dataset, which contains 13 different organisms. Results show that our miRenSVM detects more already known pre-miRNAs than *BayesMiRNAfind* : of the total 250 real pre-miRNAs, *BayesMiRNAfind* correctly predicts 220, while miRenSVM correctly predicts 233. Most of the negative training samples (~92%) used in our miRenSVM are also used to train *BayesMiRNAfind*. *BayesMiRNAfind* detects 1695 out of 1810 sequences in 3'-UTRdb and Rfam, while miRenSVM finds 1746 of the same 1810 sequences, thus miRenSVM achieves a much higher specificity.

*MicroPred* is an SVM-based method designed recently by Rukshan and Vasile to detect human miRNA gene [34]. Like miRenSVM, *microPred* uses 29 different features for SVM classification, and employs SMOTE to deal with the class imbalance problem. Although the features used in *microPred* is a little different from that in miRenSVM, they also cover the sequence, structure and thermodynamics aspects of miRNA precursors. Also trying to improve performance with an imbalance learning method, *microPred* achieves a sensitivity of little higher than our method: out of the 250 known miRNAs in miRbase12.0, *microPred* detects 236 and we detect 233. However, *microPred* predicts 516% more miRNA candidates than miRenSVM (394 compared to 64). Thus, miRenSVM has a much higher specificity than *microPred*, although *microPred* specificity is estimated high. The better performance of miRenSVM is possibly due to the features used in the classification system. Considering that a large number of pseudo stem-loop sequences have secondary structure with multi-loops, *microPred* uses only one multi-loop relevant feature (*MFEI_4_*), while miRenSVM uses four (*MFEI_4_, dP/n_loops, %*(*A-U*)*/n_loops, %*(*G-C*)*/n_loops*).

## Discussion

The miRenSVM was first trained on Homo sapiens and Anopheles gambiae genomes, and got 93.05% sensitivity, 96.5% specificity and 96.1% accuracy via outer 3-fold cross validation method. We then applied it to detect new miRNAs of *hsa* and *aga* genome in miRBase13.0. All 27 new pre-miRNAs were correctly detected. To further demonstrate the advantage of our approach, we tested miRenSVM on 27 additional animal genomes registered in miRBase13. Out of the 5238 animal pre-miRNAs across the 27 other species, miRenSVM correctly identified 4863, i.e, the recognition rate is 92.84%. The approach outperformed another recently published method [32] in detecting miRNA precursors with multi-branched loops, and obtained higher and more reliable results than the existing methods [23,25,32], while there is a little overlap among sets of miRNA candidates predicted by the different methods.

Since the number of possible candidate hairpins within the whole genome is very large and the number of real pre-miRNA is still small for some species, current specificity is still not satisfactory for multi-genomes applications and some false positive predictions can be produced. Finding more information to reduce the false positive rate should be further investigated. However, latest reports suggested that some human miRNA precursors have Box H/ACA snoRNA features [33]. It might be necessary for us to reconsider those previously regarded as false-positive predictions, since our dataset contains a certain amount of *hsa* and *aga* snoRNAs.

## Conclusion

In this study, we presented miRenSVM, a SVM-based computational approach that detects real miRNA precursors from pseudo ones with their intrinsic features. MiRenSVM uses both global and local intrinsic features of known miRNAs as its input. Several machine learning technologies including feature selection, imbalance learning and multi-classification were applied. Our approach is more general than the existing methods, since it is not sensitive to pre-miRNA's structure and thermodynamic characteristics. And it can achieve better prediction performance than the existing methods.

## Methods

### Dataset

**Figure 2:**
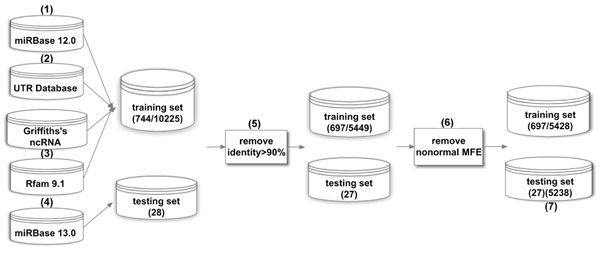
**Construction of training and testing datasets**. We built the training and testing datasets step by step. First, we collected data from five different data sources. Then, *squid*, *RNAfold* and *UNAfold* were employed to further filter the data. Finally, we constructed one training set (697 positive samples and 5428 negative samples) and two testing sets: one contains 27 bran-new *hsa* and *aga* pre-miRNA, the other contains 5238 other hairpin sequences in miRBase13.0 besides *hsa* and *aga*.

Constructing positive and negative samples sets is essential to training a machine learning classifier. It is naturally to take the already known miRNAs as the positive samples. The difficulty is to decide the best negative samples for training the classifiers. Since the number of miRNAs in a given genome is unknown [19], it is not suitable to randomly extract stem-loops sequences from the genomes. To produce high specificity in the prediction of new candidate miRNAs, the negative examples should be highly similar to the miRNA themselves. We collected negative samples in two ways: (1) Using samples from the mRNA 3'-untranslated region (3'-UTR). It has been proved that there is none predicted hsa and aga miRNA sequence in the UTRdb [23,34] . (2) Using ncRNA recognized so far including these miRNA from Rfam9.1 [35] and other datasets. The resulting dataset contains two kinds of representative species, Homo sapiens (*hsa*) and Anopheles gambiae (*aga*), both have been well studied in previous researches [23,24,36]. Construction of the dataset including both training and testing samples involves several steps. Figure 2 illustrates the process where each step is described as follows.

(1) 692 *hsa* and 52 *aga* pre-miRNA sequences in miRBase12.0 were chosen to serve as the positive set.

(2) 9225 *hsa* and 92 *aga* 3’UTR sequences in 3’-UTRdb (release 22.0) whose length ranges from 70nt and 150nt were chosen to form one part of the negative set.

(3) For *hsa*, an ncRNA dataset was already collected by Griffiths-Jones [37] that was used in [32] lately, but none sncRNA dataset of *aga* is available now. We selected all 256 *aga* ncRNA sequences in Rfam9.1, in which 68 sequences that were redundant or longer than 150nt were removed. These sequences form another part of the negative set, which are listed in the additional file 2.

(4) 14 *hsa* and 14 *aga* new hairpin sequences in miRBase13.0 were used to evaluate our miRenSVM system.

(5) In this step, sequences with the similarity score higher than 0.90 were removed by CD-HIT program [38] from the training set and testing set respectively. The 27 selected testing sequences were summarized in Table S3 of the supplementary file.

(6) 21 sequences from 3’-UTRdb, whose second structure could not be predicted by *RNAfold*[39] or *UNAfold*[40] were removed. Finally, we constructed a training set with 697 true pre-miRNA sequences, 5428 pseudo pre-miRNA sequences, and a testing set with 27 bran-new real pre-miRNA sequences. After predicting the secondary structure, nearly 84% of the 5428 pseudo miRNA precursors have the secondary structure with multi-loop.

(7) 27 animal genomes (not including *has* and *aga*) in miRBase13.0 contain 5238 pre-miRNA sequences. We collected these sequences and used them to further evaluate the proposed approach miRenSVM.

### Feature selection

The extraction of an appropriate set of features with which a classifier is trained is one of the most challenging issues in machine learning-based classifier development. In our study, both hairpin secondary structure and multi-loop structure features were considered. Concretely, we characterized a miRNA precursor by 65 local and global features that capture its sequence, secondary structure and thermodynamic properties. These features were subsumed into three groups as follows.

#### 32 triplet elements

Sequence and structure properties are characterized by triplet structure-sequence elements proposed in [25]. In the predicted secondary structure, there are only two states for each nucleotide, paired or unpaired, indicated by brackets (‘(’ or‘)’) and dots (‘.’), respectively. We do not distinguish these two situations in this work and use ‘(’ for both situations, and GU wobble pair is allowed here. For any 3 adjacent nucleotides, there are 8 possible structure compositions: ‘(((’, ‘((.’, ‘(..’, ‘(.(’, ‘.((’, ‘.(.’, ‘..(’ and ‘…’. Considering the middle nucleotide among the 3, there are 32 (8*4) possible structure-sequence combinations, which are denoted as “U(((”, “A((.”, etc.

#### 15 base pair features

Some secondary structure relevant features are already introduced by existing pre-miRNA classification methods [27,32]. In this paper, we included 11 secondary structure features (*G/C ratio, %C+G, dP, Avg_BP_Stem, Diversity, |A-U|/L, |G-C|/L, |G-U|/L,* (*A-U*)*/n_stems,* (*G-C*)*/n_stems,* (*G-U*)*/n_stems*) in our miRenSVM. Furthermore, for identifying real miRNA precursors with multi- loop, we used four new features related to the loop number in the predicted secondary structure. They are:

♦ *dP/n_loops,* where *n_loops* is the number of loops in secondary structure.

♦ *%*(*A-U*)*/n_loops, %*(*G-C*)*/n_loops, %*(*G-U*)*/n_loops,* where *%*(*X-Y*) is the ratio of X-Y base pairs in the secondary structure.

These features were extracted using the *RNAfold* program contained in Vienna RNA package (1.8.3) [39] with default parameters.

#### 18 thermodynamic features

It has been proved that using only secondary structure is not enough to effectively predict miRNA [30]. Since miRNA precursors usually have lower MFE than other small ncRNAs and random short sequences, thus MFE related features were introduced, such as (*dG, MFEI_1_, MFEI_2_, MFEI_3_, MFEI_4_, Freq*)*.* Other 8 global thermodynamics features (*NEFE, Diff, dH, dS, Tm, dH/L, dS/L, Tm/L*)*,* and 4 statistically significant features (*p-value_MFE, p-value_EFE, z-score_MFE, z-score_EFE*) were chosen from previous research [23, 24, 36]. When evaluating those statistically significant features related with MFE and ensemble free energy (EFE), for each original sequence, 300 random sequences were generated by Sean Eddy's *squid* program [30]. *dH, dS, Tm, dH/L, dS/L, Tm/L* were calculated by *UNAfold 3.7.* More detail of all the 65 features are provided in additional file 1.

We used *F*-score to measure the discriminatory power of each feature above. *F*-score is a simple technique that measures the discrimination of two sets of real numbers. Given a set of training vectors x*_k_*, *k* = 1,…, *m,* if the number of positive and negative instances are *n+* and *n_-,_* respectively, then the *F*-score of the *i*th feature is defined as:(1)

where  are the average values of the *i*th features of the whole, positive, and negative data sets, respectively;  is the *i*th feature of the *k*th positive instance, and  is the *i*th feature of the *k*th negative instance. Larger *F*-scores indicate better discrimination [41]. All the 65 local and global candidate features were ranked by *F*-score in order to determine which features will be used in the final model.

### The miRenSVM approach

#### Support vector machine

The internal of miRenSVM is Support Vector Machine, a supervised classification technique derived from the statistical learning theory of structural risk minimization principle [42]. A support vector machine constructs a hyperplane or set of hyperplanes in a high-dimensional space, which can be used for classification, regression or other tasks. SVM has been adopted extensively as an effective discriminative machine learning tool to address the miRNA prediction problem [25, 27, 43]. The model selection for SVMs involves the selection of a kernel function and its parameters that yield the optimal classification performance for a given dataset [44]. In our study, we used radial basic function (RBF) due to its higher reliability in finding optimal classification solutions in most situations. The SVM algorithm was implemented by C++ interface *libsvm* (version 2.89) package [41], and the training process of miRenSVM follows the guidelines described in [45].

#### SVM classifiers ensemble

One major factor that will influence the performance of a machine learning system is class imbalance, that is, the examples of some classes heavily outnumber the examples of the other classes [46]. Training a classifier system with an imbalance dataset will result in poor classification performance, especially for the rare classes [47]. And a classifier should have good and balanced performance over all classes for it to be useful in real-world applications.

For miRNA gene detection, the imbalance issue was widely recognized [32]. Existing machine learning based methods either employ random under-sampling to choose a portion of representative examples or just ignore it. It has already shown that both random over-sampling and random under-sampling have some drawbacks. The former does not add any information in addition to incurring large amount of compitation cost, and the later actually misses information and thus leads to poor performance. There remains a challenge: for a given dataset, how to select an appropriate sampling proportion?

In this work, the training dataset contains 697 positive (real pre-miRNA) samples and 5428 negative (pseudo pre-miRNA) samples, the ratio of negative to positive is 7.79:1. To address the drawbacks of over-sampling and under-sampling, we employed a SVM ensemble scheme. We tried to generate training sets with a desired distribution such that neither removing any training sample nor increasing the training time. An ensemble SVM classifier has several advantages over the ordinary classifiers. First, an ensemble SVM classifier exploits the information of the entire dataset, while random under-sampling uses only part of the dataset; On the other hand, it consumes less computation compared to random over-sampling. Second, an ensemble SVM classifier is able to overcome some drawbacks of a single classifier. With multi sub SVM classifiers, miRenSVM is more robust and expected to learn the exact parameters for a global optimum [42].

**Figure 3:**
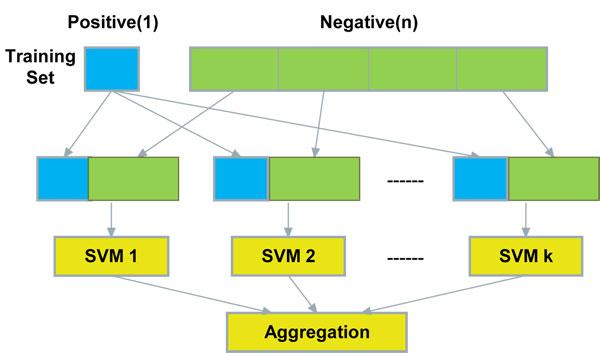
**The Architecture of miRenSVM.** The original negative samples in the training set are divided into *k* equal partitions (*k* ranges from 1 to the ratio of negative samples to positive samples). The final decision is made by aggregating the results of *k* sub-SVM classifiers that are trained by the entire positive samples and a partition of negative samples. Two aggregation methods are considered in this work: *majority vote* and *mean distance*.

Figure 3 shows the ensemble scheme of miRenSVM. Here, we used the same strategies as in [48] to sample the training dataset. First, splitting the negative examples into *k* partitions where *k* is chosen from 1 to the ratio of the majority class' size to the minority class' size. Second, generating individual subsets by combining the positive set with each partition of the negative samples. Third, training SVMs independently over every subset of the training set, and finally combining all constituent SVMs by certain of strategy to get the ensemble classifier. *Majority vote* is a widely used method to combine the results of several SVM sub-classifiers. In this paper, in addition to *majority vote*, we also used another technique called *mean distance*. Unlike *majority vote*, in the *mean distance* scheme, each sample is tested only one time by using one SVM sub-classifier. While training classifiers, we evaluated the center vector of each training set. To classify an unlabeled sample, the distance between the sample and each center vector will be calculated, and the sample will be labelled by the SVM sub-classifier whose center vector is the nearest one to the sample under testing.

### Performance evaluation method and metrics

#### Outer 3-fold cross validation

We used the *libsvm* 2.89 package to establish the miRenSVM classification system. Here, the complete training dataset is randomly divided into three equally sized partitions, while each partition has the same ratio of positive samples to negative samples. Then, any two partitions are merged together as the training dataset to train an SVM classifier. Following that, the resulting model is tested over the third data partition. This procedure is repeated three times with different combinations of training (two partitions) and testing (the remaining partition) datasets in an outer 3-fold cross validation style, and the classification result is gotten by averaging the results of the three tests above.

A straightforward way to evaluating the performance of a classifier is based on the confusion matrix. With this matrix, it is possible to evaluate a number of widely used metrics to measure the performance of a learning system, such as *accuracy* (Acc). However, Acc cannot be used to measure the performance of a classifier precisely when the class imbalance problem is present, as it does not reveal the true classification performance of the rare classes [47,49]. Therefore, in addition to Acc, we also used *sensitivity* (SE), *specificity* (SP) to evaluate the performance of a classifier. In order to exploit both positive and negative samples as much as possible, we also used their *geometric mean* (*G*m). Actually we pay more attention to *G*m than to other three metrics, as *G*m is an aggregated performance measure. These performance metrics are defined as follows:(2)

where *TP*, *FP*, *TN* and *FN* are the numbers of true positive predictions, false positive predictions, true negative predictions and false negative predictions, respectively.

## Competing interests

The authors declare that they have no competing interests.

*Funding*: This research was supported by the National Basic Research Program of China under grant no.2010CB126604.

## Authors' contributions

JD constructed the model, performed the experiments and prepared the manuscript. SG and JH guided the research and scheme design, and helped to prepare and improve the manuscript. All authors read and approved the manuscript.

## Supplementary Material

Additional file 1 Description: We surveyed pre-miRNA registered in miRBase with secondary multi-loop brunch secondary structure or with a MFE higher than -16 kal/mol, and showed the results in Table S1 and S2. Table S3 lists the 27 bran-new *hsa* and *aga* pre-miRNA sequences used as testing set. Table S4 shows the detail results of 27 other animal genomes. We also supplied some detail of the features used in the main paper Click here for file

Additional file 2 Description: An Anopheles gambiae (aga) ncRNA dataset is built by selecting sequences whose secondary structures can be predicted by *RNAfold* and *UNAfold* in Rfam9.1. Furthermore, sequences with identity higher than 90% are removed. Click here for file
