# Supplementary material for: MiRenSVM: towards better prediction of microRNA precursors using an ensemble SVM classifier with multi-loop features
Source: BMC Bioinformatics. 2010 Dec 14;11(Suppl 11):S11. doi: 10.1186/1471-2105-11-S11-S11 (PMC3024864; doi:10.1186/1471-2105-11-S11-S11)
Supplement: Additional file 1 — Description: We surveyed pre-miRNA registered in miRBase with secondary multi-loop brunch secondary structure or with a MFE higher than -16 kal/mol, and showed the results in Table S1 and S2. Table S3 lists the 27 bran-new hsa and aga pre-miRNA sequences used as testing set. Table S4 shows the detail results of 27 other animal genomes. We also supplied some detail of the features used in the main paper [file 1471-2105-11-S11-S11-S1.pdf]

Supplementary

MiRenSVM: Towards Better Prediction of MicroRNA Precursors Using an Ensemble SVM Classifier with Multi-loop Features

Jiandong Ding[1], Shuigeng Zhou[1§] and Jihong Guan[2§]

[1]Shanghai Key Lab of Intelligent Information Processing, and School of Computer Science, Fudan University, Shanghai 200433, China.

[2]Department of Computer Science and Technology, Tongji University, Shanghai 201804, China.

[§]Corresponding author

Email addresses:

Jiandong Ding: jdding@fudan.edu.cn

Shuigeng Zhou: sgzhou@fudan.edu.cn

Jihong Guan: jhguan@tongji.edu.cn

Table S1: Homo sapiens pre-miRNAs with multi-loops secondary structure [1]:

Database	Num_loops	Num_total	Proportion(%)
miRBase 5.0	14	207	6.76
miRBase 8.2	36	462	7.79
miRBase 13.0	34	715	4.76
**Total:**	**84**	**1384**	**6.07**

*Num_loops: number of *hsa* pre-miRNA with multi-loop secondary structure

*Num_total: number of *hsa* pre-miRNA in the database

Table S2: Homo sapiens pre-miRNAs in miRBase13.0 [2] whose MFE is higher than -16kal/mol (predicted by RNAfold [3] under the default parameters)

>hsa-mir-1973 MI0009983
UAUGUUCAACGGCCAUGGUAUCCUGACCGUGCAAAGGUAGCAUA
(((((((....((.(((((......)))))))....).)))))) (-10.80)
>hsa-mir-1978 MI0009988
UAGACGGGCUCACAUCACCCCAUAAACAAAUAGGUUUGGUCCUAGCCUUUCUA
((((.(((((...((((..((...........))..))))...))))).)))) (-13.70)
>hsa-mir-2052 MI0010486
CUGUUUUGAUAACAGUAAUGUCCCUUUAGUUCAAAGUUACCAGCUAUCAAAACAA
.((((((((((.(.(((((................)))))..).)))))))))). (-10.69)
>hsa-mir-2054 MI0010488
CUGUAAUAUAAAUUUAAUUUAUUCUCUAUCAUUAAAAAAUGUAUUACAG
((((((((((..((((((...........))))))...)))))))))) (-8.20)
>hsa-mir-198 MI0000240
UCAUUGGUCCAGAGGGGAGAUAGGUUCCUGUGAUUUUUCCUUCUUCUCUAUAGAAUAAAUGA
(((((..((...((((((((.(((.............)))))))))))...))...))))) (-15.44)
>hsa-mir-384 MI0001145
UGUUAAAUCAGGAAUUUUAAACAAUUCCUAGACAAUAUGUAUAAUGUUCAUAAGUCAUUCCUAGAAAUUGUUCAUAAUGCCUGUAACA
.((((...((((.(((...(((((((.((((((..((((.........)))).))).....))).)))))))...))).)))))))).<br>(-15.00)
>hsa-mir-923 MI0005715
UAUUUGUCAGCGGAGGAAAAGAAACUAACCAGGAUUCCCUCAGUAAUGGCGAGUG
(((((((((((.((((....(((.((.....)).))))))).))..))))))))) (-13.00)
>hsa-mir-924 MI0005716
AAUAGAGUCUUGUGAUGUCUUGCUUAAGGGCCAUCCAACCUAGAGUCUACAAC
..((((.(((((.((((((((.....)))).)))))))....))).)))).... (-12.30)
>hsa-mir-1279 MI0006426
AUAUUCACAAAAAUUCAUAUUGCUUCUUUCUAAUGCCAAGAAAGAAGAGUAUAAGAACUUCC
.............(((.(((..(((((((((.......)))))))))...))).)))..... (-12.10)
>hsa-mir-1308 MI0006441
CCCCGCAUGGGUGGUUCAGUGGCAGAAUUCUCAAAUUGUAAUCCCCAUAAUCCC
......(((((.((((((((...((....))...)))).)))))))))...... (-12.60)
>hsa-mir-1321 MI0006652
ACAUUAUGAAGCAAGUAUUAUUAUCCCUGUUUUACAAAUAAGGAAAUAAACUCAGGGAGGUGAAUGUGAUCAAAGAUAG
......(((.(((..((((....((((((.((((...........))))..))))))))))..)))..)))....... (-11.60)
>hsa-mir-1322 MI0006653
AGUAUCAUGAAUUAGAAACCUACUUAUUACAUAGUUUACAUAAGAAGCGUGAUGAUGCUGCUGAUGCUGUA
(((((((.(.((((...((((.(((((...........))))).)).))..))))....))))))))... (-11.30)

Table S3: Sequences of 14 *hsa* and 13 *aga* pre-miRNA whose identity is lower than 90% (predicted by CD-HIT [4])

**13 *aga* pre-miRNAs:**
>aga-mir-137 MI0010601
AAAACUUGGUUGGCCACGCGUAUUCUUGGGUUACUAACACACUGUUUAUGUUGUUAUUGCUUGAGAAUACACGUAGUUGACUAGUGUUGUA
>aga-mir-190 MI0010594
UGUUUGGUGGGACAGUUUCGGUGAGAUAUGUUUGAUAUUCUUGGUUGUUAAGAUUUCAAUUAUCACCCAGGAAUCAAACAUAUUAUUACCGUGACUGUCGU
>aga-mir-263b MI0010606
UGACCAAUUAUGGACCUUGGCACUGGGAGAAUUCACAGUGGAUCGUACCAUAUCGUUUCUGUGGAUCUUUUCGUGCCAUCGUUCAGAUUUGGUGCC
>aga-mir-286 MI0010605
GUCAUAAUUUGGGGCGAUUGUCGGACUAGUCGCUGUCGCAGUCAUUCGGUUUCACCUUAGUGACUAGACCGAACACUCGCGUCCUAAACGAACGAC
>aga-mir-309 MI0010607
AAGAUGCGACAAACUCCGUCCAGAGGGUGUCCGAUUUCAAUGAACUCAUCACUGGGCAAAGUUUGUCGCAUAAA
>aga-mir-927 MI0010596
UAGUUAAUGGUUUGUUUUAGAAUUCCUACGCUUUACCCGUGAUUAAAGUAGUGCGGCAAAGCGUUUGGAUUCUGAAACGAAACGUUAACAACG
>aga-mir-929 MI0010595
CCUACGCGUGGGAUUAAAUUGACUCUAGUAGGGAGUCCUUUUAUACGAGCGACUCCCUAACGGAGUCAGAUUGAUUCCGGUAGUGCACC
>aga-mir-957 MI0010600
UGAUCACUGCGUGCGUUAGUUUUGGGCGGGUUUUAGUGUAUUUCGAUGAGAAUUCUAUUGAAACCGUCCAAAACUGAGGCUGGCAGAUGGUUAC
>aga-mir-965-1 MI0010602
CGAAGUAAAUCGACGAAUUCUGAAAGGGGAAUGCUGUACACUCUAUGUGCUAAAAAUAUCCAUAAGCGUAUAGCUUUUCCCAUUUAGCGUUCGUUGCGAGCAAA
>aga-mir-970 MI0010599
AUGGCUGACGGAAGAUAGCCAGCGUUUGUUUUAUUUGGUAUGUUACUACUGCUGCAAGCUAUAUCAUAAGACACACGCGGCUAUUUUCCGCAACCCGA
>aga-mir-988 MI0010598
CUGUGGAGCAUUGGACAUUGCCGGUGUGUGUUUUGUGACAAUGAGAUUUAAUAGUUUAAGUUCAUCCCCUUGUUGCAAACCUCACGCUGGCGGUGUUUCCGA
>aga-mir-993 MI0010597
UGCGCUCCUCCGUGACCUACCCUGUAGUUCCGGGCUUUUGUGGGUUGAAAUAUUAUACAUAUAUAAUCUCAUAUACGCUUAUCAGAAGCUCGUUUCUAUAGAGGUAUCUCAGGGAGUGAA
>aga-mir-1000 MI0010604
CCUAGCAGUCGAUGAUAUUGUCCUGUCACAGCAGUACUUUUUGCCUAGUUUACUGUUGUUUCGGGACAUUUCCAUCGACGCUAGGGUUUUCAU
**14 *has* pre-miRNAs:**
>hsa-mir-1204 MI0006337
ACCUCGUGGCCUGGUCUCCAUUAUUUGAGAUGAGUUACAUCUUGGAGGUGAGGACGUGCCUCGUGGU
>hsa-mir-1972 MI0009982
UAUAGGCAUGUGCCACCACACCUGGCUUAAAUGUGUCAUUUAAAAAUUCAGGCCAGGCACAGUGGCUCAUGCCUGUA

>hsa-mir-1973 MI0009983
UAUGUUCAACGGCCAUGGUAUCCUGACCGUGCAAAGGUAGCAUA
>hsa-mir-1974 MI0009984
UGUUCUUGUAGUUGAAAUACAACGAUGGUUUUUCAUAUCAUUGGUCGUGGUUGUAGUCCGUGCGAGAAUA
>hsa-mir-1975 MI0009985
AGUUGGUCCGAGUGUUGUGGGUUAUUGUUAAGUUGAUUUAACAUUGUCUCCCCCCACAACCGCGCUUGACUAGCU
>hsa-mir-1976 MI0009986
GCAGCAAGGAAGGCAGGGGUCCUAAGGUGUGUCCUCCUGCCCUCCUUGCUGU
>hsa-mir-1977 MI0009987
UUGAUUAGGGUGCUUAGCUGUUAACUAAGUGUUUGUGGGUUUAAGUCCCAUUGGUCUAGUAAGGGCUUAGCUUAAUUAA
>hsa-mir-1978 MI0009988
UAGACGGGCUCACAUCACCCCAUAAACAAAUAGGUUUGGUCCUAGCCUUUCUA
>hsa-mir-1979 MI0009989
UCUUUACUCCCACUGCUUCACUUGACUAGCCUUUAAAAAAGAAAGGCUUGGUUUGAUGAAUGGGUGAGAGAAAAGG
>hsa-mir-2052 MI0010486
CUGUUUUGAUAACAGUAAUGUCCCUUUAGUUCAAAGUUACCAGCUAUCAAAACAA
>hsa-mir-2053 MI0010487
CUUGCCAUGUAAAUACAGAUUUAAUUAACAUUUGCAACCUGUGAAGAUGCAAAACUUUAA
>hsa-mir-2054 MI0010488
CUGUAAUAUAAAUUUAAUUUAUUCUCUAUCAUUAAAAAAUGUAUUACAG
>hsa-mir-2110 MI0010629
CAGGGGUUUGGGGAAACGGCCGCUGAGUGAGGCGUCGGCUGUGUUUCUCACCGCGGUCUUUUCCUCCCACUCUUG
>hsa-mir-2113 MI0003939
UUUUCAAAGCAAUGUGUGACAGGUACAGGGACAAAUCCCGUUAAUAAGUAAGAGGAUUUGUGCUUGGCUCUGUCACAUGCCACUUUGAAAA

Table S4: Results of miRenSVM on animal pre-miRNAs published in miRBase13.0.

**Species (Animal)**	**Evaluated pre-miRNAs**	**Correctly Predicted**	**Accuracy (%)**
Apis mellifera	64	60	93.75
Branchiostoma floridae	74	62	83.78
Bombyx mori	61	58	95.08
Bos taurus	359	341	94.99
Capitella sp. I	72	59	81.94
Caenorhabditis briggsae	98	89	90.82
Caenorhabditis elegans	155	148	95.48
Canis familiaris	325	317	97.54
Ciona intestinalis	25	25	100
Ciona savignyi	27	26	96.30
Drosophila melanogaster	152	138	90.79
Drosophila pseudoobscura	73	70	95.89
Danio rerio	337	331	98.22
Fugu rubripes	133	132	99.25
Gallus gallus	471	437	92.78
Lottia gigantea	57	46	80.70
Monodelphis domestica	119	115	96.64
Macaca mulatta	458	417	91.05
Mus musculus	568	521	91.73
Nematostella vectensis	49	43	87.76
Ornithorhynchus anatinus	344	307	89.24
Pan troglodytes	599	528	88.15
Rattus norvegicus	286	271	94.76
Schmidtea mediterranea	79	75	94.94
Sus scrofa	55	54	98.18
Tribolium castaneum	55	53	96.36
Tetraodon nigroviridis	143	140	97.90
Total	***5238***	***4863***	***92.84***

Features:

Triplet element (32):

Triplet structure-sequence element is proposed by Xue *et.al* [5]. The detail of these features has already been well described in the main article.

Base pair feature (15):

11 of these features have been used in previous miRNA gene predicting methods [6, 7]

Four new features relevant to loop number in the predicted secondary structure are introduced:

- *dP/n_loops*, where n_loops is the number of loops in secondary structure.
- *%(A-U)/n_loops*, *%(G-C)/n_loops*, *%(G-U)/n_loops*, where %(X-Y) is the ratio of X-Y base pairs in the secondary structure.

Thermodynamics features (18):

6 MFE related features; 8 other global thermodynamics features and 4 statistically significant features are chosen from previous research [6, 7, 8].

The definitions of other features already used by existing pre-miRNA classification methods are available in [7]'s supplementary data.

References:

1. Jiang P, Wu H, Wang W, et al.: **MiPred: classification of real and pseudo microRNA precursors using random forest prediction model with combined features.** *Nucleic acids research* 2007, **35**:W339-44.

2. Griffiths-Jones S, Saini HK, van Dongen S, Enright AJ: **miRBase: tools for microRNA genomics.** *Nucleic acids research* 2008, **36**:D154-8.

3. Hofacker IL: **Vienna RNA secondary structure server.** *Nucleic acids research* 2003, **31**:3429-31.

4. Li W, Godzik A: **Cd-hit: a fast program for clustering and comparing large sets of protein or nucleotide sequences.** *Bioinformatics* 2006, **22**:1658-9.

5. Xue C, Li F, He T, et al.: **Classification of real and pseudo microRNA precursors using local structure-sequence features and support vector machine.** *BMC bioinformatics* 2005, **6**:310.

6. Ng KL, Mishra SK: **De novo SVM classification of precursor microRNAs from genomic pseudo hairpins using global and intrinsic folding measures.** *Bioinformatics* 2007, **23**:1321-30.

7. Batuwita R, Palade V: **microPred: effective classification of pre-miRNAs for human miRNA gene prediction.** *Bioinformatics* 2009, **25**:989-95.

8. Freyhult E, Gardner PP, Moulton V: **A comparison of RNA folding measures.** *BMC bioinformatics* 2005, **6**:241.
